# Living life precariously with rheumatoid arthritis - a mega-ethnography of nine qualitative evidence syntheses

**DOI:** 10.1186/s41927-018-0049-0

**Published:** 2019-02-06

**Authors:** Fran Toye, Kate Seers, Karen Louise Barker

**Affiliations:** 1 Nuffield Orthopaedic Centre, Oxford University Hospitals NHS Foundation Trust, Oxford, UK; 2 Nuffield Department of Orthopaedics, Rheumatology and Musculoskeletal Sciences, University of Oxford, Oxford, UK; 3 Warwick Research in Nursing, Warwick Medical School, University of Warwick, Coventry, UK

**Keywords:** Qualitative research, Qualitative evidence synthesis, Rheumatoid arthritis, Patient experience, Systematic review, Meta-ethnography

## Abstract

**Background:**

Rheumatoid arthritis is an autoimmune disease that causes joint inflammation. It affects around 400,000 people in the UK and 1 million adults in the USA. Given the appropriate treatment, many can have relatively few symptoms. It is therefore important to understand what it is like to live with rheumatoid arthritis and gain insight into peoples’ decisions about utilising healthcare. The aims of this study were: (1) to bring together qualitative evidence syntheses that explore patients’ experience of living with rheumatoid arthritis and (2) develop a conceptual understanding of what it is like to live with rheumatoid arthritis.

**Methods:**

We used the methods of mega-ethnography. The innovation of mega-ethnography is to use conceptual findings from qualitative evidence syntheses as primary data. We searched four bibliographic databases from inception until September 2018 to identify qualitative evidence syntheses that explored patients’ experience of rheumatoid arthritis.

**Results:**

We identified 373 qualitative evidence syntheses, removed 179 duplicates and screened 194 full text studies. We identified 42 qualitative evidence syntheses that explored the experience of pain or arthritis and 9 of these explored the experience of rheumatoid arthritis. We abstracted ideas into 10 conceptual categories: (1) rheumatoid arthritis is in control of my body (2) rheumatoid arthritis alters reciprocity; (3) rheumatoid arthritis is an emotional challenge; (4) rheumatoid arthritis disrupts my present and future self; (5) the challenge of balancing personal and work life; (6) I am trying to make sense of what is happening; (7) rheumatoid arthritis is variable and unpredictable; (8) rheumatoid arthritis is invisible; (9) I need a positive experience of healthcare, and (10) I need to reframe the situation. We developed a conceptual model underpinned by *living life precariously with rheumatoid arthritis*.

**Conclusions:**

This is the second *mega*-ethnography, or synthesis of qualitative evidence syntheses using the methods of meta-ethnography. Future research should consider the proliferation of qualitative evidence synthesis in order to avoid duplication of research effort. Our model for rheumatoid arthritis has some important clinical implications that might be transferable to other musculoskeletal conditions.

**Electronic supplementary material:**

The online version of this article (10.1186/s41927-018-0049-0) contains supplementary material, which is available to authorized users.

## Background

Rheumatoid Arthritis (RA) is an autoimmune disease that causes joint inflammation. Arthritis Research UK estimates that RA affects around 400,000 people in the UK [1], with about 3 times more women than men affected. In the USA, prevalence of RA is estimated as 0.53–0.55%, which accounted for more than a million adults in 2014 [2]. Symptoms can vary from mild joint pain and swelling to severe disease with extensive pain and disability in 5% of people. Given the appropriate treatment, many can have relatively few symptoms. It is therefore important to understand what it is like to live with RA in order to gain insight into peoples’ decisions about utilising healthcare.

The number of qualitative evidence syntheses (QES) is increasing [3–5] and in some areas, several QES’s explore the same or similar topics. Toye and colleagues highlight the danger of research duplication but also suggest that ‘framed in a more positive light, this increase might provide an opportunity for useful synthetic products for the purposes of policy and practice’ [6]. Frost and colleagues also explore the possibility of syntheses of QES [7]. As part of their Global Year of Excellence in Pain Education 2018, the International Association for the Study of Pain (IASP) invited FT to lead a team to produce a resource to help educators include qualitative research in education [8]. As part of this resource, we aimed to identify qualitative evidence syntheses (QES) in the field of pain and summarise their findings. This study brings together the QES that explored patients’ experience of living with RA and provides a conceptual understanding of what it is like. We explore the differences in the experience of living with RA and other forms of non-specific chronic pain as reported in a previous mega-ethnography [6].

## Methods

We used the methods of mega-ethnography reported by Toye, Seers and Barker in the first ever *mega-ethnography* of what it is like to live with chronic pain [6]. Mega-ethnography aims to synthesise the findings from existing QES; it is a review of reviews. Mega-ethnography uses the stages of meta-ethnography [9] and aims to offer conceptual insight that it greater than the sum of the constituent QES. Although there have been calls to standardise the reporting of QES [10–12], and suggestions for appraising confidence in QES [13–21], there are currently no agreed methods for this.

We aimed to identify any QES that explored the experience of living with pain or arthritis. Arthritic conditions had not been included in the QES of chronic musculoskeletal pain by Toye and colleagues [22]. We searched four bibliographic databases (Medline, Cinahl, Psychinfo, Embase) from inception until November 2017. We used the search terms shown in Table 1. FT screened the titles, abstracts and full text of potential studies for relevance and included QES that explored patients’ experience of RA. Wee ran the search again to check that there were no additional QES exploring the experience of RA published between November 2017 and September 2018.

**Table 1:** Example of search terms used for PsychInfo

QES terms	(“qualitative evidence synthesis”).ti,ab
	(QES).ti,ab
	(metasynthes* OR meta-synthes* OR “meta synthesis”).ti,ab
	(metasummar* OR meta-summar* OR “meta summary”).ti,ab
	(metastud* OR meta-stud* OR “meta study”).ti,ab
	(metaethnog* OR meta-ethnog* OR “meta ethnography”).ti,ab
	(“critical interpretive synthesis”).ti,ab
	(“realist synthesis”).ti,ab
	(“thematic synthesis”).ti,ab
	(qualitative ADJ4 systematic*).ti,ab
	(qualitative ADJ4 review).ti,ab
	(qualitative ADJ4 synthes*).ti,ab
	(noblit ADJ4 hare).ti,ab
Combined with	exp PAIN/ (pain).ti,ab exp. ARTHRITIS/ (arthritis).ti,ab
Limits	none

The ‘data’ of *mega*-ethnography are QES findings. Some methods of QES (including meta-ethnography) abstract their individual findings into a line of argument or model. However, this is not standard amongst QES approaches. For this study, we used individual QES findings, and not their lines of argument, as data. FT and KB read the reviews in alphabetical order to identify their findings. They made ‘a list of key metaphors, phrases, ideas and/or concepts’ [9] (page 28) so that they could compare these across studies. KB and FT challenged each other’s interpretation of the QES findings in order to remain confident that their interpretation remained grounded in that study [23]. Any disagreement was discussed and resolved with a third reviewer (KS). Once all reviewers had agreed upon a description of each QES finding, FT re-wrote this finding in the first person. We have found that writing concepts in the first person is a powerful way for readers (and reviewers) to fully engage in the meaning and sentiment of each concept. It also facilitates the use of accessible language for a diverse audience.

Once we had a list of the QES findings written in first person, FT, KS and KB ‘translated’ [9] into each other by comparing concepts, observing similarities and differences and gradually organising them into conceptual categories. This process of ‘constant comparison’ and abstraction is integral to qualitative research methods [24]. In other meta-ethnographies [3], researchers have used an ‘index’ paper as a way of ‘orienting the synthesis’ [25]. We did not use an index paper as this can potentially have a dramatic effect on the final interpretation [26].All three reviewers organised categories collaboratively through discussion. The final analytic stage of meta-ethnography, ‘Synthesising translations’ involves organising categories into an ‘interpretive order’ or model. We intended to produce a line of argument by developing ‘a grounded theory that puts the similarities and differences between studies into interpretive order’ [9] (page 64). Through constant comparison we compared similarities and differences in order to find ‘the essence of an idea that can extend beyond its constituent parts’ [6].

## Results

**Fig. 1:**
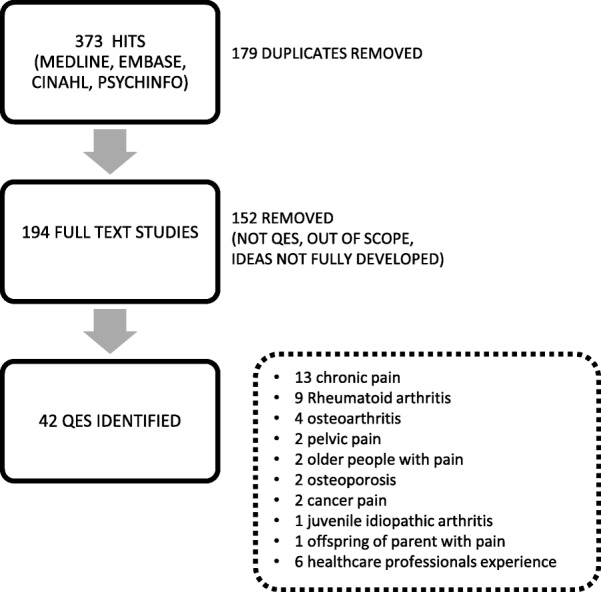
Flowchart of search process

**Fig. 2:**
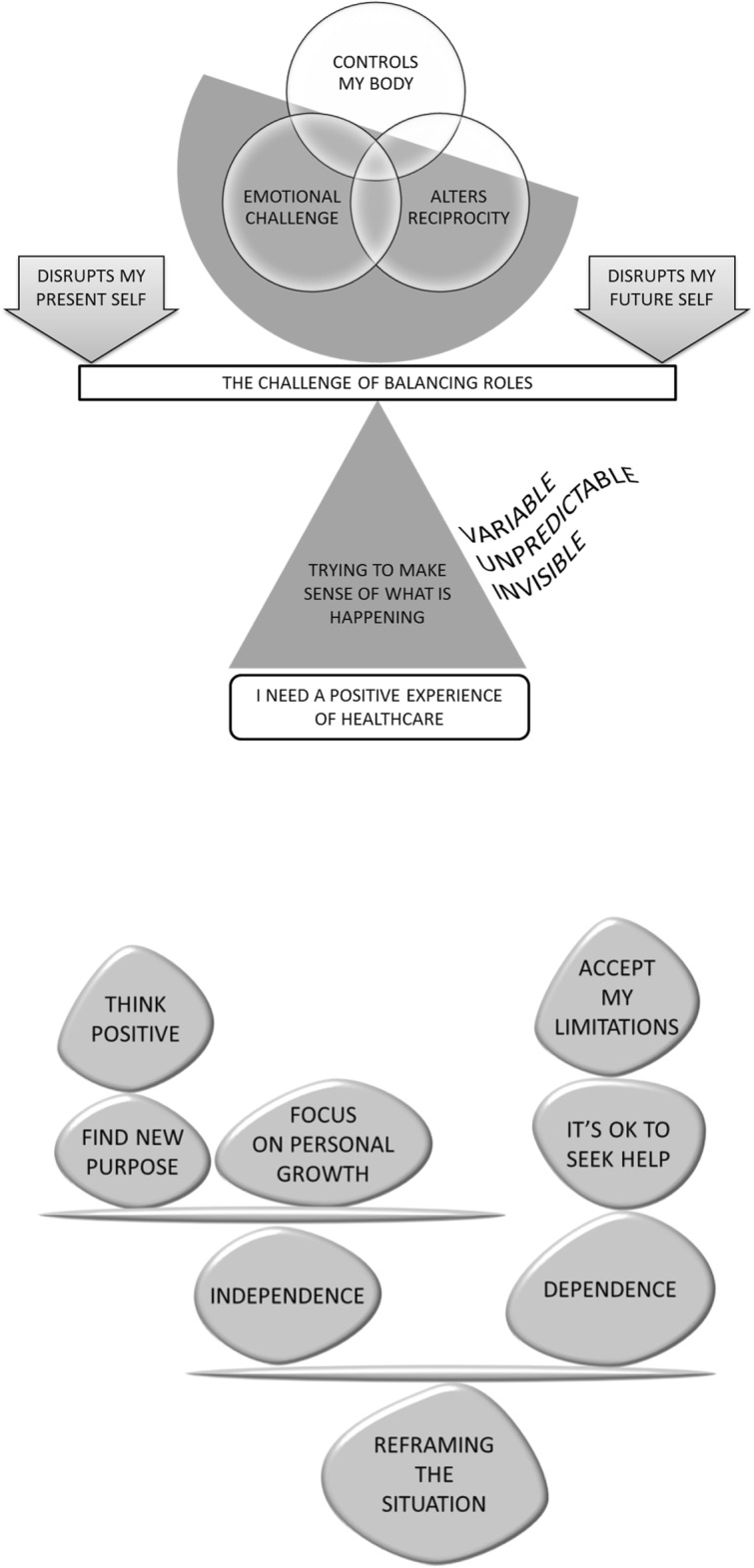
Conceptual model: (**a**) living precariously with rheumatoid arthritis & (**b**) reframing the situation. Presents our conceptual model: (**a**) Rheumatoid arthritis controls my body and alters the reciprocity of my relationships. It is an emotional challenge and balancing roles is precarious. Rheumatoid arthritis disrupts who I am and my vision for the future. I try to make sense of what is happening but my condition is unpredictable, variable, and sometimes invisible. A positive experience of healthcare would give some stability in this precarious situation. (**b**) Reframing the situation and living well with rheumatoid arthritis means finding a balance between independence and dependence: accepting the body’s imitations and realising that it is OK to seek and accept help. Focus on personal growth, think positively and find purpose

We identified 373 potential QES from 4 medical databases (Medline, Embase, Cinahl, Psychinfo). We removed 179 duplicates. We considered the full text of 194 studies and removed 152 studies because; they were not QES, they were out of scope, or we felt that the ideas were not fully developed. We identified 42 QES that explored the experience of pain and/or arthritis (chronic non-malignant pain (including fibromyalgia) [22, 27–38], pelvic pain [39, 40], osteoarthritis [41–44], older people’s experience of pain [45, 46], osteoporosis [47, 48], juvenile idiopathic arthritis [49], cancer pain and living with cancer after treatment [50, 51], offspring’s experience of living with a parent with chronic pain [52], healthcare professionals’ experience of treating people with chronic pain [53–58] and RA [59–67]. Figure 1 presents a flowchart of our search process. We found no additional QES on RA published between November 2017 and September 2018.These nine studies, including findings from 128 published reports and more than 2000 people with RA, are listed in Additional file 1. Table 2 shows the author, year, aim of study, method of QES, number of primary studies included, number of findings and countries included in the original QES. We extracted 76 review findings from the nine QES. These findings are available in a resource that we produced for the International Association for the Study of Pain (IASP) Global year of Education 2018 [8] We abstracted the 76 findings into 10 conceptual categories: (1) RA is in control of my body (2) RA alters reciprocity; (3) RA is an emotional challenge; (4) RA disrupts my present and future self; (5) the challenge of balancing personal and work life; (6) I am trying to make sense of what is happening; (7) RA is variable and unpredictable; (8) RA is invisible; (9) I need a positive experience of healthcare, and (10) I need to reframe the situation. Table 3 shows which QES supported each of these categories. We describe each conceptual category and illustrate each with two examples of QES findings that support it. QES findings are described in the first person: these descriptions are not direct quotations from the original QES. We also describe a line of argument which synthesises our conceptual categories into a whole (Fig. 2). Kelly and colleagues uniquely explore patients’ experiences of Disease Modifying Anti-Rheumatic Drugs (DMARDS) in RA and spondyloarthritis [66] . Although the experience of taking DMARDs may be integral to the experience of RA we did not synthesise these findings into our conceptual model, but have summarised them in Table 4. This will allow the reader to consider and draw on the concepts from that study.

**Table 2:** Author, year, aim, method of QES, number of participants, and number of concepts included

Author, year	Aim of study	Analytic method	Number of primary studies	Analytic output	Countries included
Campbell & colleagues 2011 [64]	To explore experiences related to the aetiology, treatment, management and lived experience of rheumatoid	META-ETHNOGRAPHY	25	6 themes	13 UK, 7 USA, 2 Canada, 1 Denmark, 1 Sweden, 1 New Zealand
Daker-white, donovan & campbell 2014 [63]^a^	To synthesize published qualitative studies concerning the lived experience of rheumatoid arthritis	META-ETHNOGRAPHY	40	4 themes	Not specified in original article
Fedderson& colleagues 2017 [62]	To derive new conceptual understanding about how women with rheumatoid arthritis manage their illness, motherhood and paid work	META-SYNTHESIS	6	4 themes	2 Denmark, 1 USA, 1 UK, I Canada, 1 Sweden
Hoving & colleagues 2013 [61]	To summarize qualitative studies that explore experiences of patients with inflammatory arthritis to remain employed or return to work	THEMATIC ANALYSIS	10	6 themes	3 UK, 3 Netherlands, 1 Canada, 1 USA, 1 Sweden, 1 Ireland
Hulen & colleagues 2016 [67]	To identify needs, goals and expectations of rheumatoid arthritis patients	GROUNDED THEORY	13	3 themes	9 UK, 3 Holland, 2 Japan, 2 Sweden, 1 Canada, 1 Norway, 1 China, Japan and USA
Kelly & colleagues 2017 [66]	To describe patients’ experiences of Disease Modifying Anti-Rheumatic Drugs (DMARDS) in rheumatoid arthritis and spondyloarthritis	THEMATIC SYNTHESIS	55	25 subthemes (6 themes)	25 UK, 5 USA, 5 Netherlands, 5 Canada, 4 Sweden, 2 Australia, 2 Belgium, 1 Austria, 1 Denmark, 1 Germany, 1 Ireland, 1 Turkey, 1 Norway, 1 Netherlands, Austria and UK
Lin & colleagues 2011 [65]	To describe the status of spiritual well-being in patients with rheumatoid arthritis	META-SUMMARY	10	18 subthemes (4 themes)	4 UK, 3 USA, 1 Canada, 1 South Korea, 1 Sweden
Stack & colleagues 2011 [60] ^a^	To explore the drivers of and barriers to help-seeking behaviour in people with a new onset of rheumatoid arthritis.	GROUNDED THEORY	21	5 themes	8 UK, 6 Canada, 4 USA, 1 Australia, 1 South Africa, 1 Sweden
Stack & colleagues 2013 [59]	To identify the earliest symptoms associated with the onset of rheumatoid arthritis	GROUNDED THEORY	26	5 themes	11 UK, 6 USA, 4 Canada, 1 Australia, 1 Austria, 1 South Korea, 1 South Africa

^a^Numbers of studies obtained by direct author correspondence as lacked clarity in original study

**Table 3:** QES supporting each conceptual category

Theme	Campbell & colleagues 2011 [64]	Daker-white, Donovan & Campbell 2014 [63]	Fedderson& Colleagues 2017 [62]	Hoving & colleagues 2013 [61]	Hulen & colleagues 2016 [67]	Kelly & colleagues 2017 [66]	Lin & colleagues 2011 [65]	Stack & colleagues 2011 [60]	Stack & colleagues 2013 [59]
Rheumatoid arthritis is in control of my body	X	X		X	X	Studies explore experience of Disease modifying anti-rheumatic drugs (DMARDS)		X	X
Rheumatoid arthritis alters reciprocity	X	X	X	X	X				
Rheumatoid arthritis is an emotional challenge		X	X	X					X
The challenge of balancing roles			X	X					
rheumatoid arthritis disrupts mx present and future self			X	X	X				
rheumatoid arthritis is variable and unpredictable	X	X	X	X					X
rheumatoid arthritis is invisible	X	X		X				X	
i am trying to make sense of what is happening	X							X	X
i need a positive experience of healthcare				X			X		
Reframing the situation is precarious	X	X			X		X

**Table 4:** Disease modifying anti-rheumatic drugs (DMARDS) – Kelly & Colleagues 2017 [66]

Maintaining control	Patients wanted full information about DMARDs so that they could make their own choice. Some were prepared to accept complications and would take extreme risks.
Distressing uncertainties & consequences	Some were worried about the safety of DMARDs and were uncertain about their efficacy: ‘My orthopaedist said: “arthritis patients actually have two diseases, that is arthritis and methotrexate”; I have always remembered that.’
Negotiating treatment expectations	Emotional responses to DMARDs hinged on impact, or expected impact of medication. For some Biologic DMARDs were seen as the last hope. Some were disappointed with the effects; for others, the effects exceeded hopes.
Powerful social influences	Family, friends, doctors and nurses could have a strong influence on the decision to take DMARDS. Patients needed to have confidence in the doctor, yet experience of healthcare was variable.
Privilege and right of access to biologics	Some felt it was a privilege to take biologic DMARDS and could feel guilty. Others felt that everyone with RA had a right to be prescribed. Some were very worried about DMARDs being withdrawn and hid any side effects.
Intensifying disease identity	Some were shocked about being prescribed ‘strong’ medications and felt this was a sign of increasing disease severity. Some felt dependent on lifelong medication which made them contemplate the incurability of RA.

This table summarise the findings from Kelly and colleagues ‘Patients’ attitudes and experiences of disease-modifying anti-rheumatic drugs in rheumatoid arthritis and spondyloarthritis: A qualitative synthesis’ [66]

### Rheumatoid arthritis is in control of my body

The conceptual category *RA is in control of my body* [67, 59–61, 63, 64] describes a feeling that the symptoms of RA control me. It incorporates a wide range of bodily symptoms from RA: pain, fatigue, loss of energy, stiffness, deformity, lost concentration and poor sleep. Some described how their bodily appearance could exacerbate suffering.

CAMPBELL & COLLEAGUES 2011 [64]: Living with the symptoms of rheumatoid arthritis: Pain dominates. My symptoms are variable and unpredictable. I don’t know from one day to the next whether I will be feeling better, worse or the same. I don’t know which part of my body it will affect. Pain is the only certainty. Pain brings fatigue lowers my personal reserve. I dread becoming dependent on others. There are no signs that verify my suffering to others. Pain and fatigue are ambiguous as they are invisible. My swollen and disfigured body adds to my suffering.

HOVING & COLLEAGUES 2013 [61]: Disease symptoms and effects on work: I am tired and lack energy. I am in pain. My joints are stiff and I am physically limited. I can’t concentrate or solve problems. Fatigue is particularly difficult for me and other people don’t acknowledge it. RA is invisible and that makes it worse. My symptoms fluctuate and this makes it difficult to plan.

### Rheumatoid arthritis alters reciprocity

The conceptual category *RA alters reciprocity* [67, 61–64] describes how RA alters the balance of reciprocal social and work relationships and can cause an uncomfortable imbalance. This imbalance gradually erodes people’s normal roles, work, social and family life. The category incorporates feeling guilty for becoming a burden; it describes needing help but not wanting to ask for help; it describes lost independence and yet, at the same time, lost intimacy.

DAKER-WHITE, DONOVAN & CAMPBELL 2014 [63]: the nature of symptoms and strategic responses: I have been redefined by RA. RA gives me pain. It makes my joints stiff and causes immobility. I suffer with fatigue. However symptoms are fluctuating and unpredictable on a day-to-day basis. My normal roles are changed or lost. I feel like a social outcast. I can become emotional at times. It permeates “every sphere of life”. I have lost some independence and fear dependency.

FEDDERSON & COLLEAGUES 2017 [62]: social interactions in the performance of three interdependent sub-identities – motherhood: It was a huge pressure when my children were young. I didn’t think that I should have children because of RA. Being pregnant might have made it worse. I might have had to stop my medication as it may have been harmful. RA might be passed on to the child. Now my partner has had to take over household tasks. I feel like a burden but I am grateful for help. I feel guilty because I can’t do things.

### Rheumatoid arthritis is an emotional challenge

The category RA *is an emotional challenge* [59, 61–63] describes the emotional impact of RA that permeates every sphere of life. Some felt low, angry, guilty, worried and afraid. Some described thoughts of ending things.

HOVING & COLLEAGUES 2013 [61]: Emotional challenges: RA is an emotional challenge. I am afraid, anxious and uncertain about the future. I feel sad about my limitations. I feel dependent and helpless. I feel guilty if I take time off work. I feel uneasy about asking for help. I am a burden to the organization. My aspirations are not being fulfilled. I will no longer take risks. I find it difficult to set boundaries. My frustration and low mood can make it difficult to deal with colleagues and create a pleasant disposition at work.

DAKER-WHITE, DONOVAN & CAMPBELL 2014 [63]: the nature of symptoms and strategic responses: I have been redefined by RA. RA gives me pain. It makes my joints stiff and causes immobility. I suffer with fatigue. However symptoms are fluctuating and unpredictable on a day-to-day basis. My normal roles are changed or lost. I feel like a social outcast. I can become emotional at times. It permeates “every sphere of life”. I have lost some independence and fear dependency.

### The challenge of balancing roles

The *challenge of balancing roles* [61, 62] describes a struggle to balance family, work, social and personal roles. This struggle can leave no reserve for personal fulfilment or fun. It describes the need to prioritise in order to deal with the unpredictability of symptoms. Challenging days in one domain can spill over into other domain. At times one role will need to take precedence over another. However, trying to maintain one role can have a detrimental effect on another.

FEDDERSON& COLLEAGUES 2017 [62]: social interactions in the performance of three interdependent sub-identities: It is a challenge to balance work, motherhood and RA. If there is too much pressure in one area it affects the others. If pressure is off in one area, I feel some relief in the other areas. I need flexibility in these areas to stay in control.

HOVING & COLLEAGUES 2013 [61]: Interpersonal issues and choices affecting work and family life: I need my colleagues to know and understand about my RA. If you don’t tell other people it is difficult for others to understand and give the necessary support. Communication is vital to understanding and support. Difficulties at work can spill over into home life. Trying to stay at work can be detrimental to my personal life.

### Rheumatoid arthritis disrupts my present and future self

*RA disrupts my present and future self* [67, 61, 63] describes the biographical disruption of RA and feelings of lost identity. It describes how RA changes what a person is now and what they planned to be in the future.

CAMPBELL & COLLEAGUES 2011 [64]: Consequences for identity and problems maintaining taken-for-granted activities: I can no longer do ‘taken-for-granted’ normal activities. My biography has been disrupted by RA. It assaults my body and disrupts my social life. I have lost my previous life and work role. I can no longer take things for granted. RA has challenged the normal reciprocity in my life. The uncertainly of RA means that I have to continuously monitor my symptoms and manage them. I can no longer take anything for granted. Life is now fraught with danger. Even ‘everyday objects and events take on an alarming character’ and make me feel insecure. I am locked into my house.

DAKER-WHITE 2014 [63]: the nature of symptoms and strategic responses: I have been redefined by RA. RA gives me pain. It makes my joints stiff and causes immobility. I suffer with fatigue. However symptoms are fluctuating and unpredictable on a day-to-day basis. My normal roles are changed or lost. I feel like a social outcast. I can become emotional at times. It permeates “every sphere of life”. I have lost some independence and fear dependency.

### Rheumatoid arthritis is variable and unpredictable

This describes the ambiguity of RA and its *variable and unpredictable* symptoms [59, 61–64]. People with RA do not know what each day will bring. The category includes the dread of a future with worsening symptoms and the need to continuously monitor the body. Life has become fraught with danger and normal functions cannot be taken for granted.

CAMPBELL & COLLEAGUES 2011 [64]: Living with the symptoms of rheumatoid arthritis: Pain dominates. My symptoms are variable and unpredictable. I don’t know from one day to the next whether I will be feeling better, worse or the same. I don’t know which part of my body it will affect. Pain is the only certainty. Pain brings fatigue lowers my personal reserve. I dread becoming dependent on others. There are no signs that verify my suffering to others. Pain and fatigue are ambiguous as they are invisible. My swollen and disfigured body adds to my suffering.

FEDDERSON& COLLEAGUES 2017 [62]: social interactions in the performance of three interdependent sub-identities: Living with RA: The amount that I focus on RA is linked to how severe my symptoms are at the time. The unpredictability of fatigue, the fluctuating course and progression of RA is the greatest challenge to my role at work and as a mother. I try to plan and prioritise so that I can deal will unpredictability and be prepared for bad days. I have not personal reserve for leisure or fun. At times, I try to ignore it, even though I know that it can make things worse.

### Rheumatoid arthritis is invisible

*RA is invisible* [60, 61, 63, 64] describes the challenge of living with invisible symptoms and the frustration when others don’t take your suffering seriously. However, because it is invisible some manage to hide it from others in an attempt to hold onto a positive sense of self-identity.

STACK & COLLEAGUES 2011 [60]: Speaking to others, gathering information and seeking alternative treatments: It has come on very slowly so there is no need to rush to the doctor. I ask around to try and find an explanation for my symptoms. I ask my family and friends before I go to the doctor. My partner insisted that I seek help. My friend said that I might have RA. My friends have suggested different remedies so I haven’t gone to the doctor yet. I feel a bit isolated because people are not taking it seriously. I hide it from family and friends because they will think I am a ‘moaner’. People are not very empathetic. My RA is invisible so I don’t have to tell people.

DAKER-WHITE, DONOVAN & CAMPBELL 2014 [63]: the body in rheumatoid arthritis: I don't want people to see my distorted body. How I look affects how I feel. At times I feel absolutely rubbish because I look awful. I hate my body. I avoid going out. However, in many ways RA is an invisible illness and it is frustrating and upsetting because others cannot appreciate my suffering.

### I am trying to make sense of what is happening

The conceptual category *I am trying to make sense of what is happening* [59, 60, 64] describes the process of making sense of RA symptoms. When symptoms started, people used common-sense explanations to make sense of them. For some people, the diagnosis of RA brought relief. For others, sudden onset caused fear. Insidious and gradual onset increased peoples’ uncertainty about what action to take; is it just normal aches and pains? Has it been passed down to me? Some prioritised more ‘serious’ conditions until things got too much for them to cope with. Some felt that society did not take joint problems very seriously.

STACK 2011& COLLEAGUES [60]: Making sense of early symptom experience and prototypes of RA: I didn’t know anything about RA before I got it and may have gone to the doctor earlier. Everyone knows about cancer and heart disease. They don’t think joint problems are serious. Is it actually an illness and what do I need to do; can anything be done? It has been creeping up for ages so I don’t know what it is. Is it just old people that get it? Is it caused by ‘wear and tear’? I don’t need to seek help. Is it caused by stress in my life; work; injury; childbirth; overdoing it? It might just be temporary and I don’t need help. Is it related to my other illness or osteoarthritis? I didn’t think it was RA. I have no family history so it can’t be that

CAMPBELL & COLLEAGUES 2011 [64]: Lay perceptions of the causes of arthritis: I need to understand why this is happening to feel a sense of order from fragmentation and loss of control. Is it the weather, my age, my injury, diet, infection, bursitis, my shoes, air pollution. Is it my age; is it my body breaking down?

### I need a positive experience of healthcare

This conceptual category describes the *need for a positive experience of healthcare* [67, 60]. At times patients feel that their healthcare professionals might judge them badly or dismiss their symptoms; they don’t want to waste anyone’s time.

STACK & COLLEAGUES 2011 [60]: Accessing health services and attitudes towards healthcare professionals: I don’t have confidence in my healthcare professional’s competence and knowledge so I don’t want to go. They might blame me for my illness because of my lifestyle. I am a bit overweight and like a drink. They might dismiss my symptoms *or* make me feel guilty or like I am a ‘hypochondriac’. Also I don’t want to waste their time. Some people go quickly to the doctor when they have symptoms; they may have had better experiences.

HULEN & COLLEAGUES 2016 [67]: Interpersonal and healthcare system interactions: I am in and out of healthcare and health care professional are an important part of my life now. I need this experience to be a positive one.

### Reframing the situation is precarious

Reframing the situation *is precarious* [65, 67, 63, 64] . It incorporates the challenge of balancing dependence and independence: accepting the limitations of my body, and learning that it is ok to seek, and accept, helps from other people, but simultaneously holding onto a sense of independence and autonomy.

LIN & COLLEAGUES 2011 [65]: Living With the Disease - 2. Accepting limitations and changes in roles: have accepted and am now calmer about changes in my work and life roles. At times I have stretched the limits of what I can do, especially at work, so that I can keep my job. I have needed to adjust my expectations and standards. It can be hard to accept limitations and its effect on normal reciprocal relationships.

DAKER-WHITE, DONOVAN & CAMPBELL 2014 [63]: biographical issues – re-defining “normal” life: My biography has been disrupted. I am struggling to adapt to my changing circumstances. My life story has three chapters: 1. the normal or past life; 2. my body out of sync; 3. finding a new way to live. Getting used to it. Adapting, or mastering RA, takes time. It is a biographical process. RA might bring some positive opportunities, for example it had made me rethink my obligations.

Reframing the situation also describes the benefits of focusing on the positive. For example, be altruistic and make a social contribution. This can give meaning and purpose to suffering. Be inspired by those less fortunate. Care for others. Focus on the purposes in life, like family. Focus on personal growth. Don’t worry so much about what others think.

LIN & COLLEAGUES 2011 [65]: Reframing the Situation - 1. Changing values: It helps to think differently about my self-worth: Don’t think that you are a failure because you can’t do the things you used to do; think about the positive changes in your life; focus on personal growth through adversity; think positively about yourself and your body; don’t worry about what others think’ focus on your personal needs.

HULEN & COLLEAGUES 2016 [67]: Achieving normalcy and maintaining wellness: Going to work and joining in socially is integral to my identity. It is important to my sense of well-being to find a way to live normally and appear normal to others. Important to maintain well-being and find a way to live normally. I want to ‘forget’ about having RA and enjoy life. I want to do the things that I want and stay in control. I want to be confident and motivated. I want to accept and find meaning in illness. I want to cope with its emotional impact, improve my mood and reduce stress or depression.

## Conceptual model

Our conceptual model hinges on living life precariously with RA. Firstly, living with the symptoms of RA: it controls the person’s body, significantly alters reciprocal relationships and is an emotional challenge. Balancing roles becomes precarious. RA disrupts my self now and my vision for the future. I try to make sense of what is happening but the unpredictability, variability and (sometimes) invisibility of RA can make this difficult. A positive experience of healthcare would provide some stability in this precarious situation. Secondly, reframing the situation and trying to live well with RA involves finding a balance between independence and dependence: accepting the bodies limitations and realising that it is OK to seek and accept help, yet at the same time focusing on personal growth, thinking positively and finding new purpose.

## Discussion

Our mega-ethnography synthesises concepts from nine QES which draw on findings from 128 published qualitative studies. Mega-ethnography, and other forms of review or mega-review, can make a large body of qualitative research available for practice, policy and education. This may become increasingly important at the number of QES is increasing [5, 68]. We aimed to synthesise QES that explored patients’ experience of living with RA and develop a conceptual understanding using the methods of *mega-ethnography.* Central to our model is the concept of a precarious existence. We all live life precariously: ‘Anything living can be expunged at will or by accident; and its persistence is in no sense guaranteed [69]. We considered using the word ‘*precarity’* to describe our concept. For Butler, the concept of ‘precarity’ takes us further than *precariousness. It describes a condition where* ‘certain [*marginalised*] populations … become differentially exposed’ to harm. Although for Butler, this is a politically invoked condition, its relevance for health and social care is that it infers an ethical responsibility. Precarity may be a useful concept in healthcare to help us to understand what it is like to live life marginalised by ill-health and pain.

Findings resonate with those of Toye and colleagues in a mega-ethnography of eleven QES exploring what it is like to live with chronic non-malignant pain [6]. They describe a life that is ‘impoverished and confined’ where the person with chronic pain struggles against their own body to keep hold of their sense of identity. Our findings also suggest that people living with RA feel that their altered body has taken control, and that normal roles and identities are eroded. Toye and colleagues have also described a negative experience of the healthcare system for patients with chronic non-malignant pain [22]. Similarly, our findings show that at times patients with RA can feel that their healthcare professionals might judge them badly or dismiss their symptoms. Both studies demonstrate that a positive experience of healthcare can help the process of adjusting to a chronic condition and that this cannot be underestimated.

Our model for RA also highlights some key differences between the experience of those living with RA and those living with chronic pain. Firstly, although people living with RA try to make sense of their symptoms, our findings do not support the intensity or *imperative* of the diagnostic ‘holy grail’ [6], nor the sense of lost personal credibility that accompanies absence of medical diagnosis. However, findings demonstrate that people living with RA also face the challenge of living with invisible symptoms, and frustration when others fail to appreciate personal suffering. Secondly, whereas learning to live with chronic pain hinged on letting go of the quest for the ‘holy grail’ of diagnosis [6] (which can be extremely difficult to do), our findings indicate that reframing the situation for RA involves the challenge of finding a balance between independence and a sense of encroaching dependence.

FT undertook the screening process and a second researcher did not verify the screening for the following reasons: (1) qualitative research methods do not hinge upon statistical analysis of an entire data set [3]; (2) this is a review of reviews and these are easy to recognise; (3) FT is a researcher with experience of QES and an in-depth knowledge of this topic area with the expertise to recognise relevant studies; (4) we have used this approach successfully in the first published mega-ethnography of chronic pain [6]; (5) conceptual reviews of qualitative research, such as mega-ethnography focus on conceptual analysis. We feel that, in reviews of this scale, research time is more productively spent on completing insightful analysis, and creatively disseminating findings, rather than on attempting to identify all the relevant studies. Research to explore whether or not there is any added value from verification in qualitative research strategies could help to ensure that funded research time was used more productively.

This is a review of QES and there is no checklist for formally appraising the quality of QES. Although there have been calls to standardise the reporting of QES [10–12], qualitative analysis is underpinned by an interpretive framework. Although, for quantitative reviews of the effectiveness of interventions, there is now an established method for evaluating confidence in review (http://gradeworkinggroup.org/), attempts to establish confidence in QES findings are in their infancy [13, 14]. There are two proposed methods for determining confidence in QES: ‘Confidence in the Evidence from Reviews of Qualitative Research’ (GRADE-CERQual) [15–21], and ‘Confidence in the output of qualitative research synthesis’ (ConQual) [13]. Both of these regard the quality of included primary studies to be important if readers are to be confident in a review finding. However, one of the challenges is that there is very limited agreement about what determines a ‘good’ primary qualitative study [26, 70–72]. Some feel that insightful studies might be excluded if we focus on methodological reporting [3, 26]. Hence, a significant number of qualitative reviewers choose not to quality appraise [3, 5]. Methodological appraisal does not help us to appraise the relative value or contribution of QES findings. Even though conceptual insight underpins the aims of meta-ethnography, this facet of quality is not considered in either GRADE-CERQual or ConQual. For the purposes of QES ‘… time might be better spent abstracting concepts rather than pettifogging over fine details of [methodological] appraisal, particularly when there is no agreed method for determining what good quality is [73] [page 58]. A full discussion of the challenges of determining confidence in QES can be found in the National Institute for Health Research Journals Library [73]. For the purposes of this study, all reviewers collaboratively extracted ideas from each QES. Further research to consider the value of quality appraisal for conceptual reviews would be useful.

Meta-ethnography is a form of QES that incorporates a line of argument or conceptual model that is, by its very nature, an interpretation. Not all QES include a conceptual model. The intention of meta-ethnography is to bring together conceptual categories into a line of argument that is greater than, and therefore *different to*, the sum of its individual parts. These conceptual models can therefore go beyond the constituent elements [6, 22, 27, 47, 73, 74]. This interpretation is the end product of a rigorous research process. We would encourage readers to use and develop our suggested conceptual model in line with their own experience.

One of the criticisms of large QES is that it is possible to lose sight of the context and nuances of the original primary studies. This criticism is potentially amplified in a *mega*-QES where data goes through a further level of abstraction. It would be unrealistic to claim that our interpretations were not influenced by our own existing ideas. However, we made every effort to keep an open mind and use existing concepts to sensitise us to new insights, rather than to define our interpretation: ‘definitive concepts provide prescriptions of what to see, sensitising concepts merely suggest directions along which to look’ [75](p. 7). Although qualitative research emphasises the unique experience, we also argue that it can say something valuable beyond its context.

## Conclusion

The innovation of this study is to present a mega-ethnography of QES to develop our conceptual understanding of what it is like to live with RA. Our model for RA has some important clinical implications: It is important that healthcare professionals recognise that it is precarious to live with RA and the profound impact on the person with RA in terms of identity, family, social and work life, both now and in the future. It would be useful for these professionals to contemplate the encroaching sense of dependence that is part of the experience of living with RA. Interventions that focus on the personal and emotional impact of RA might help to facilitate a person’s capacity to reframe the situation and live well with RA.

## Additional file

Additional file 1: Studies included in each QES, number of participants and condition. This additional file provides a list of studies in included in each of the 9 qualitative evidence syntheses, along with the number of participants and health condition. (DOCX 159 kb)

## Abbreviations

QES: Qualitative Evidence Synthesis
RA: Rheumatoid arthritis
