# Supplementary material for: Living life precariously with rheumatoid arthritis - a mega-ethnography of nine qualitative evidence syntheses
Source: BMC Rheumatol. 2019 Feb 6;3:5. doi: 10.1186/s41927-018-0049-0 (PMC6390589; doi:10.1186/s41927-018-0049-0)
Supplement: Supplementary file 1 — Studies included in each QES, number of participants and condition. This additional file provides a list of studies in included in each of the 9 qualitative evidence syntheses, along with the number of participants and health condition. (DOCX 159 kb) [file 41927_2018_49_MOESM1_ESM.docx]

**Additional file 1: Studies included in each QES, number or participants and condition**

| AUTHOR, YEAR | CAMPBELL & COLLEAGUES  2011[64] | DAKER-WHITE, DONOVAN & CAMPBELL 2014[63]^a^ | FEDDERSON& COLLEAGUES  2017[62] | HOVING & COLLEAGUES  2013[61] | HULEN & COLLEAGUES  2016[60] | KELLY & COLLEAGUES  2017[59] | LIN & COLLEAGUES  2011[58] | STACK & COLLEAGUES  2011[57]^a^ | STACK & COLLEAGUES  2013[56] | NUMBER OR PARTICIPANTS AND CONDITION |
| --- | --- | --- | --- | --- | --- | --- | --- | --- | --- | --- |
| AHLMEN ET AL. 2005[[1](#_ENREF_1)] |  | y |  |  | Y | y |  |  |  | 25 RA |
| ARCHENHOLTZ ET AL. 1999 [[2](#_ENREF_2)] | Y | y |  |  |  |  |  |  |  | 50 RA and 50 SLE |
| ASHE ET AL. 2005 [[3](#_ENREF_3)] |  |  |  |  |  |  |  | y |  | 10 RA |
| BACKMAN ET AL. 2007[[4](#_ENREF_4)] |  | y |  |  |  | y |  |  | y | 12 RA, AS, JIA, SLE |
| BARLOW ET AL. 2001 [[5](#_ENREF_5)] ^b^ |  |  |  | Y |  |  |  |  |  | 6 AS |
| BATH ET AL. 1999 [[6](#_ENREF_6)] |  |  |  |  |  | y |  |  |  | 15 RA |
| BERGSTEN ET AL. 2011[[7](#_ENREF_7)] |  |  |  |  | Y |  |  |  |  | 16 RA |
| BERNATSKY ET AL. 2010 [[8](#_ENREF_8)] |  |  |  |  | Y |  |  | y | y | 18 RA, 40 HCPs |
| BJØRNER AND HANSEN, 1993 [[9](#_ENREF_9)] | Y | y |  |  |  |  |  |  |  | NK |
| BOONEN ET AL. 2009 [[10](#_ENREF_10)] ^b^ |  |  |  |  |  | y |  |  |  | 19 AS |
| BROWN AND WILLIAMS 1995 [[11](#_ENREF_11)] | Y | y |  |  |  |  | Y | y | y | 7 RA |
| BUITINGA ET AL. 2012[[12](#_ENREF_12)] |  |  |  |  | Y |  |  |  |  | 16 RA |
| BURY, 1982 [[13](#_ENREF_13)] | Y | y |  |  |  |  |  | y | y | 25 RA |
| BURY, 1988 [[14](#_ENREF_14)] | Y | y |  |  |  |  |  | Y | y | NK |
| CARR ET AL. 2003[[15](#_ENREF_15)] |  |  |  |  | Y |  |  |  |  | 39 RA |
| CHILTON 2008[[[16](#_ENREF_16)] |  |  |  |  |  | y |  |  |  | 8 RA |
| CINAR 2014 [[17](#_ENREF_17)]b |  |  |  |  |  | y |  |  |  | Not qualitative |
| COADY 2007[[18](#_ENREF_18)] |  | y |  |  |  |  |  |  |  | 8 RA |
| CODD ET AL.2010 [[19](#_ENREF_19)] |  |  |  | Y |  |  |  |  |  | 10 RA |
| DETAILLE ET AL.2003 [[20](#_ENREF_20)] |  |  |  | Y |  |  |  |  |  | 21 RA, 23 diabetes, 25 hearing loss |
| DILDY, 1996 [[21](#_ENREF_21)] | Y | y |  |  |  |  |  |  | y | RA 14 14 |
| DONOVAN [[22](#_ENREF_22)] |  |  |  |  |  | y |  |  |  | 30, RA, 9 other IA , 15 OA |
| DONOVAN ET AL. 1989 [[23](#_ENREF_23)] | Y | y |  |  |  |  |  |  |  | Not qualitative |
| DONOVAN, 1991 [[24](#_ENREF_24)] | Y |  |  |  |  |  |  |  |  | 30, RA, 9 other IA , 15 OA |
| DUBOULOZ ET AL 2004[[25](#_ENREF_25)] |  | y |  |  |  |  | y |  |  | 6 RA |
| EDWARDS 2004 [[26](#_ENREF_26)] |  |  |  |  |  | y |  |  |  | 7 RA |
| FAIR 2003 [[27](#_ENREF_27)] |  |  |  |  |  |  |  |  | y | 17 RA & 5 HCPs |
| FELDTHUSEN ET AL. 2013[[28](#_ENREF_28)] |  |  | Y |  |  |  |  |  |  | 25 RA |
| FLUREY 2014 [[29](#_ENREF_29)] |  |  |  |  |  | y |  |  |  | 30 RA |
| FLUREY 2014 [[30](#_ENREF_30)] |  |  |  |  |  | y |  |  |  | 15 RA |
| FRAENKEL 2015 ([[31](#_ENREF_31)] |  |  |  |  |  | y |  |  |  | 88 RA |
| GARCIAPOPA-LISEANU 2005 [[32](#_ENREF_32)] |  |  |  |  |  | y |  |  |  | 22 SLE and 18 RA |
| GILWORTH ET AL. 2001 [[33](#_ENREF_33)] | Y | y |  | Y |  |  |  |  |  | 47 RA and 2 employers |
| GOODACRE & GOODACRE 2004[[34](#_ENREF_34)] |  | y |  |  |  | y |  |  |  | 29 RA |
| GRANT 2001 [[35](#_ENREF_35)] | Y | y |  |  |  |  |  |  |  | 2 RA, 1 SLE, 1 reactive arthritis |
| GRIFFITH AND CARR 2001 [[36](#_ENREF_36)] |  |  |  |  |  |  |  | y | y | Not qualitative |
| GRONNING 2011 [[37](#_ENREF_37)] |  |  |  |  |  | y |  |  |  | 26 RA, psoriatic arthritis or polyarthritis |
| HAY ET AL 2008 [[38](#_ENREF_38)] |  |  |  |  |  |  |  | y |  | Not qualitative |
| HEADLAND 2006 [[39](#_ENREF_39)] |  |  |  |  |  | y |  |  |  | 18 arthritis, 2 carers |
| HEWLETT ET AL, 2005 [[40](#_ENREF_40)] |  | y |  |  |  |  |  |  | y | 15 RA |
| HIRSH 2009 [[41](#_ENREF_41)] |  |  |  |  |  | y |  |  |  | 27 RA |
| HOFMANN ET AL. 2015[[42](#_ENREF_42)] |  |  |  |  | Y | y |  |  |  | 17 RA |
| HOOPER ET AL 2004[[43](#_ENREF_43)] |  |  |  |  |  |  | y |  |  | 10 RA |
| HOWDEN ET AL. (2003[[44](#_ENREF_44)] |  |  |  | Y |  |  |  |  |  | 3 RA |
| HWANG, KIM & JUN 2004 [[45](#_ENREF_45)] |  |  |  |  |  |  | y |  | y | 5 RA |
| IAQUINTA AND LARRABEE 2004 [[46](#_ENREF_46)] |  | y |  |  |  |  | y |  |  | 6 RA |
| KETT 2010 [[47](#_ENREF_47)] |  |  |  |  |  | y |  |  |  | 21 RA |
| KRISTIANSEN ET AL. 2012 [[48](#_ENREF_48)] |  |  | Y |  | Y | y |  |  |  | 32 RA |
| KRISTIANSEN ET AL. 2012[[49](#_ENREF_49)] |  |  | Y |  | Y |  |  |  |  | 11 RA |
| KUMAR 2011 [[50](#_ENREF_50)] |  |  |  |  |  | y |  |  |  | 32 RA and SLE |
| KUMAR ET AL, 2010 [[51](#_ENREF_51)] |  |  |  |  |  |  |  | y | y | 10 RA |
| LACAILLE ET AL. 2007[[52](#_ENREF_52)] |  |  |  | Y |  |  |  |  |  | 36 IA (75% rheumatoid arthritis) |
| LAMBERT AND BUTIN 2000 [[53](#_ENREF_53)] | Y |  |  |  |  |  |  |  |  | 12 arthritis 14 HCPs |
| LARSSON 2009 [[54](#_ENREF_54)] |  |  |  |  |  | y |  |  |  | 20 RA |
| LEMPP 2012[[55](#_ENREF_55)] |  |  |  |  |  | y |  |  |  | 18 RA |
| LEMPP ET AL. 2006 [[56](#_ENREF_56)] |  | y |  |  |  | y |  |  | y | 26 RA |
| LI 2014 [[57](#_ENREF_57)] |  |  |  |  |  | y |  |  |  | 30 RA |
| LI ET AL. 2009 [[58](#_ENREF_58)] |  |  |  |  |  |  |  | y |  | conference abstract |
| LINBLAD 2002 [[59](#_ENREF_59)] |  |  |  |  |  | y |  |  |  | 22 RA |
| LINDEN 2010 [[60](#_ENREF_60)] |  |  |  |  |  | y |  |  |  | 11 RA |
| LOCKER 1983 [[61](#_ENREF_61)] | Y | y |  |  |  |  |  |  |  | 24 RA |
| LORISH 1990 [[62](#_ENREF_62)] |  |  |  |  |  | y |  |  |  | Not qualitative |
| LUTZE AND ARCHENHOLT 2007[[63](#_ENREF_63)] |  |  |  |  |  |  | y |  |  | 23 RA |
| MANCUSO ET AL. 2000 [[64](#_ENREF_64)] |  |  |  | Y |  |  |  |  |  | 22 RA |
| MARKUSSE 2014 [[65](#_ENREF_65)] |  |  |  |  |  | y |  |  |  | 20 RA |
| MARSHALL 2004 [[66](#_ENREF_66)] |  |  |  |  |  | y |  |  |  | 19 RA |
| MCARTHUR 2015 [[67](#_ENREF_67)] |  |  |  |  |  | y |  |  |  | 19 RA, 8 AS |
| MCPHERSON ET AL. 2001[[68](#_ENREF_68)] | Y | y |  |  |  |  | Y |  |  | 10 RA |
| MCPHERSON ET AL. 2004 [[69](#_ENREF_69)] |  | y |  |  |  |  |  |  |  | 10 RA, 10 stroke, 10 Chronic pain |
| MEADE 2013 [[70](#_ENREF_70)] |  |  |  |  |  | y |  |  |  | 14 RA |
| MEYFROIDT 2015 [[71](#_ENREF_71)] |  |  |  |  |  | y |  |  |  | 26 RA |
| MINNOCK 2016 [[72](#_ENREF_72)] |  |  |  |  |  | y |  |  |  | 10 RA |
| MITTON, TREHARN ET AL 2007[[73](#_ENREF_73)] |  |  |  |  |  |  | y |  |  | 7 RA |
| MOSS, 1997 [[74](#_ENREF_74)] | Y | y |  |  |  |  |  |  |  | 25 arthritis |
| NEILL, 2002 [[75](#_ENREF_75)] |  | y |  |  |  |  |  | y | y | 3 RA |
| NILSSON ET AL. (2007)[[76](#_ENREF_76)] |  |  |  | Y |  |  |  |  |  | 10 RA |
| NOTA 2015 [[77](#_ENREF_77)] |  |  |  |  |  | y |  |  |  | 28 RA, 3 AS 1 PA |
| NYMAN AND LUTZEN, 1999[[78](#_ENREF_78)] |  |  |  |  |  |  |  | y | y | 6 RA |
| OHARE 2000 [[79](#_ENREF_79)] |  |  |  |  |  | y |  |  |  | 18 RA |
| OLIVER ET AL. 2008)[[80](#_ENREF_80)] |  |  | Y |  |  |  |  | y | y | 22 RA |
| PASMA 2015 [[81](#_ENREF_81)] |  |  |  |  |  | y |  |  |  | 33 IA |
| PINDER, 1995 [[82](#_ENREF_82)] | Y | y |  |  |  |  |  |  |  | 9 JIA, 11 RA, 1 SLE, 1 psoriatic arthritis, 3 OA |
| PLACH ET AL. 2004 [[83](#_ENREF_83)] |  | y | Y |  |  |  |  |  |  | 20 RA |
| PLACH ET AL. 2004[[84](#_ENREF_84)] |  | y |  |  |  |  |  |  |  | 20 RA |
| RADFORD ET AL. 2008[[85](#_ENREF_85)] |  | y |  |  | Y |  |  |  |  | 12 RA |
| RAO ET AL., 1998 [[86](#_ENREF_86)] | Y | y |  |  |  |  |  |  |  | 33 RA |
| RICE AND YOUNG, 1994 [[87](#_ENREF_87)] | Y |  |  |  |  |  |  |  |  | NK |
| ROSE 2006 [[88](#_ENREF_88)] |  |  |  |  |  | y |  |  |  | 5 RA |
| RYAN & HASSELL 2003[[89](#_ENREF_89)] |  | y |  |  |  |  |  |  |  | 40 RA |
| RYAN, 1996 [[90](#_ENREF_90)] | Y |  |  |  |  |  |  |  |  | 40 RA |
| SAKALYS 1997 [[91](#_ENREF_91)] |  |  |  |  |  |  |  | y | y | 50 RA |
| SALT & PEDEN, 2011[[92](#_ENREF_92)] |  |  |  |  | Y | y |  |  |  | 30 RA |
| SALT 2012 [[93](#_ENREF_93)] |  |  |  |  |  | y |  |  |  | 15 RA |
| SANDERSON 2009 [[94](#_ENREF_94)] |  |  |  |  |  | y |  |  |  | 17 RA |
| SANDERSON 2011 [[95](#_ENREF_95)] |  |  |  |  |  | y |  |  |  | 23 RA |
| SANDERSON ET AL. 2012 [[96](#_ENREF_96)] |  |  |  |  |  | y |  |  |  | 26 RA |
| SANDERSON ET AL. 2010A[[97](#_ENREF_97)] |  |  |  |  | Y | y |  |  |  | 23 RA |
| SANDERSON ET AL. 2010B[[98](#_ENREF_98)] |  |  |  |  | Y | y |  |  |  | 23 RA |
| SANDHU 2013 [[99](#_ENREF_99)] |  |  |  |  |  | y |  |  |  | Not qualitative |
| SCHILDMANN 2008 [[100](#_ENREF_100)] |  |  |  |  |  | y |  |  |  | 22 RA |
| SCHNEIDER ET AL. 2008 [[101](#_ENREF_101)] |  | y |  |  |  |  |  | y | y | 60 RA |
| SHARIFF 2008[[102](#_ENREF_102)] |  | y |  |  |  |  |  |  |  | 46 RA |
| SHAUL 1997[[103](#_ENREF_103)] |  |  |  |  |  |  | y | y | y | 30 RA |
| SHAUL 1995 [[104](#_ENREF_104)] | Y | y |  |  |  |  |  | y | y | 30 RA |
| SHEPPARD ET AL. 2008 [[105](#_ENREF_105)] |  | y |  |  |  |  |  | y | y | 24 RA |
| SINCLAIR & BLACKBURN 2008[[106](#_ENREF_106)] |  |  |  |  |  |  | y |  |  | 19 RA |
| STAMM 2010 [[107](#_ENREF_107)] |  |  |  |  |  | y |  |  |  | 15 RA |
| STAMM ET AL. 2004[[108](#_ENREF_108)] |  | y |  |  |  |  |  |  |  | 9 RA |
| STAMM ET AL. 2008 (39) [[109](#_ENREF_109)] |  | y |  |  |  |  |  |  | y | 10 RA |
| STENSTRÖM ET AL. 1993 [[110](#_ENREF_110)] | Y | y |  |  |  |  |  |  |  | 9 RA |
| STEPHENS AND YOSHIDA 1999 [[111](#_ENREF_111)] | Y | y | Y |  |  |  |  |  |  | Not qualitative |
| STOCKDALE 2009 [[112](#_ENREF_112)] ^b^ |  |  |  |  |  | y |  |  |  | 8 AS |
| STOCKDALE 2014 [[113](#_ENREF_113)] ^b^ |  |  |  |  |  | y |  |  |  | 22 As |
| TOWNSEND 2013 [[114](#_ENREF_114)] |  |  |  |  |  | y |  |  |  | 37 RA |
| TOWNSEND ET AL. 2010 [[115](#_ENREF_115)]) |  |  |  |  |  |  |  | y | y | 8 RA |
| VAN DER ELST 2015 [[116](#_ENREF_116)] |  |  |  |  |  | y |  |  |  | 26 RA |
| VAN DER MEER ET AL. (2011[[117](#_ENREF_117)] |  |  |  | Y |  |  |  |  |  | 14 RA |
| VAN DER TUYL 2008 [[118](#_ENREF_118)] |  |  |  |  | Y | y |  |  |  | 12 RA, 15 HCPs |
| VAN DER TUYL 2015 [[119](#_ENREF_119)] |  |  |  | y |  | y |  |  |  | 47 RA |
| VAREKAMP ET AL. (2005[[120](#_ENREF_120)] |  |  |  | Y |  |  |  |  |  | 21 and 17 hcps |
| WARD ET AL. 2007 [[121](#_ENREF_121)] |  | y |  |  |  |  |  |  |  | 25 RA |
| WIENER 1975 [[122](#_ENREF_122)] | Y | y |  |  |  |  |  |  | y | 21 RA |
| WILLIAMS AND BARLOW 1998 [[123](#_ENREF_123)] | Y | y |  |  |  |  |  |  |  | 14 RA |
| WILLIAMS AND GRAHAM 2012[[124](#_ENREF_124)] |  |  |  |  |  |  |  |  | y | 22 RA |
| WILLIAMS AND WOOD [[125](#_ENREF_125)] | Y | y |  |  |  |  |  |  |  | NK |
| WILLIAMS 1984[[126](#_ENREF_126)] | Y |  |  |  |  |  |  | y | y | 30 RA |
| YOSHIDA, 1996 [[127](#_ENREF_127)] |  |  |  |  |  |  |  | y | y | 80 SCI and RA |
| ZHANG AND VERHOEF 2002 [[128](#_ENREF_128)] |  |  |  |  |  | y |  | y |  | 8 RA, 11 OA |

1. List of included studies from author [personal correspondence]
2. Sample explored AS not RA

NK = SAMPLE NOT KNOWN

SLE Systemic lupus erythematosus

AS Ankylosing Spondylitis

JIA Juvenile Idiopathic Arthritis

IA Inflammatory Arthritis

OA Osteoarthritis

1. Ahlmén M, Nordenskiöld U, Archenholtz B, Thyberg I, Rönnqvist R, Lindén L, Andersson AK, Mannerkorpi K: **Rheumatology outcomes: the patient's perspective. A multicentre focus group interview study of Swedish rheumatoid arthritis patients**. *Rheumatology* 2005, **44**(1):105-110.

2. Archenholtz B, Burckhardt CS, Segesten K: **Quality of life of women with systemic lupus erythematosus or rheumatoid arthritis: Domains of importance and dissatisfaction**. *Quality of Life Research* 1999, **8**(5):411-416.

3. Ashe B, Taylor M, Dubouloz CJ: **The process of change: listening to transformation in meaning perspectives of adults in arthritis health education groups**. *Can J Occup Ther* 2005, **72**(5):280-288.

4. Backman C, SmithdelFabro L, Smith S, Montie P, Suot M: **Experiences of mothers living with inflammatory arthritis**. *Arthritis Care & Research* 2007, **57**(3):381-388.

5. Barlow J, Wright C, Williams B, Keat A: **Work disability among people with ankylosing spondylitis**. *Arthritis Care & Research* 2001, **45**(5):424-429.

6. Bath J, Hooper J, Steel D, Reed E, Giles M, Woodland J: **Patient perceptions of rheumatoid arthritis**. *Nursing Standard* 1999, **14**(3):35-38.

7. Bergsten U, Bergman S, Fridlund B, Arvidsson B: **"Striving for a good life" - the management of rheumatoid arthritis as experienced by patients**. *The open nursing journal* 2011, **5**:95-101.

8. Bernatsky S, Feldman D, De Civita M, Haggerty J, Tousignant P, Legare J, Zummer M, Meagher T, Mill C, Roper M *et al*: **Optimal care for rheumatoid arthritis: a focus group study**. *Clinical rheumatology* 2010, **29**(6):645-657.

9. Bjørner J, Hansen I: **Reumatoid artrit og samliv: en kvalitativundersøgelse (Rheumatoid arthritis and partner relationships:a qualitative study) [Danish]**. *Nordisk Sexol* 1993, **11**:101-111.

10. Boonen A, van Berkel M, Cieza A, Stucki G, van der Heijde D: **Which Aspects of Functioning Are Relevant for Patients with Ankylosing Spondylitis: Results of Focus Group Interviews**. *The Journal of Rheumatology* 2009, **36**(11):2501.

11. Shona B, Anne W: **Women's experiences of rheumatoid arthritis**. *Journal of Advanced Nursing* 1995, **21**(4):695-701.

12. Buitinga L, Braakman-Jansen LM, Taal E, van de Laar MA: **Future expectations and worst-case future scenarios of patients with rheumatoid arthritis: a focus group study**. *Musculoskeletal Care* 2012, **10**(4):240-247.

13. Bury M: **Chronic illness as biographical disruption**. *Sociology of Health & Illness* 1982, **4**(2):167-182.

14. Bury M: **Meanings at risk: the experience of arthritis.** In: *Living with chronic illness: the experience of patients and their families.* edn. Edited by Anderson R BM. London:: Unwin Hyman; 1988: 89-116.

15. Carr A, Hewlett S, Hughes R, Mitchell H, Ryan S, Carr M, Kirwan J: **Rheumatology outcomes: the patient's perspective**. *J Rheumatol* 2003, **30**(4):880-883.

16. Chilton F, Collett R: **Treatment choices, preferences and decision‐making by patients with rheumatoid arthritis**. *Musculoskeletal Care* 2008, **6**(1):1-14.

17. Cinar FI, Cinar M, Yilmaz S, Simsek I, Erdem H, Pay S: **Thoughts and perceptions of ankylosing spondylitis patients with regard to TNF inhibitors**. *Rheumatology International* 2014, **34**(7):979-986.

18. Coady D, Armitage C, D W: **Rheumatoid arthritis patients' experiences of night pain**. *J Clin Rheumatol* 2007, **13**(2):66-69.

19. Codd Y, Stapleton T, Veale DJ, FitzGerald O, Bresnihan B: **A qualitative study of work participation in early rheumatoid arthritis**. *International Journal of Therapy and Rehabilitation* 2010, **17**(1):24-33.

20. Detaille SI, Haafkens JA, van Dijk FJ: **What employees with rheumatoid arthritis, diabetes mellitus and hearing loss need to cope at work**. *Scand J Work Environ Health* 2003, **29**(2):134-142.

21. Dildy SP: **Suffering in people with rheumatoid arthritis**. *Applied Nursing Research* 1996, **9**(4):177-183.

22. Donovan JL, Blake DR: **Patient non-compliance: Deviance or reasoned decision-making?** *Social Science & Medicine* 1992, **34**(5):507-513.

23. Donovan JL, Blake DR, Fleming WG: **THE PATIENT IS NOT A BLANK SHEET: LAY BELIEFS AND THEIR RELEVANCE TO PATIENT EDUCATION**. *Rheumatology* 1989, **28**(1):58-61.

24. Donovan J: **Patient education and the consultation: the importance of lay beliefs**. *Annals of the rheumatic diseases* 1991, **50**:418-421.

25. Dubouloz C-J, Laporte D, Hall M, Ashe B, Smith CD: **Transformation of Meaning Perspectives in Clients With Rheumatoid Arthritis**. *American Journal of Occupational Therapy* 2004, **58**(4):398-407.

26. Edwards J: **An exploration of patients' experiences of anti‐TNF therapy**. *Musculoskeletal Care* 2004, **2**(1):40-50.

27. Fair BS: **Contrasts in patients' and providers' explanations of rheumatoid arthritis**. *Journal of nursing scholarship : an official publication of Sigma Theta Tau International Honor Society of Nursing* 2003, **35**(4):339-344.

28. Feldthusen C, Bjork M, Forsblad-d'Elia H, Mannerkorpi K: **Perception, consequences, communication, and strategies for handling fatigue in persons with rheumatoid arthritis of working age--a focus group study**. *Clinical rheumatology* 2013, **32**(5):557-566.

29. Flurey CA, Morris M, Pollock J, Richards P, Hughes R, Hewlett S: **A Q-methodology study of flare help-seeking behaviours and different experiences of daily life in rheumatoid arthritis**. *BMC Musculoskeletal Disorders* 2014, **15**(1):364.

30. Flurey CA, Morris M, Richards P, Hughes R, Hewlett S: **It's like a juggling act: rheumatoid arthritis patient perspectives on daily life and flare while on current treatment regimes**. *Rheumatology (Oxford)* 2014, **53**(4):696-703.

31. Fraenkel L, Seng EK, Cunningham M, Mattocks K: **Understanding how patients (vs physicians) approach the decision to escalate treatment: a proposed conceptual model**. *Rheumatology (Oxford)* 2015, **54**(2):278-285.

32. Garcia Popa-Lisseanu MG, Greisinger A, Richardson M, O'Malley KJ, Janssen NM, Marcus DM, Tagore J, Suarez-Almazor ME: **Determinants of treatment adherence in ethnically diverse, economically disadvantaged patients with rheumatic disease**. *J Rheumatol* 2005, **32**(5):913-919.

33. Gilworth G, Woodhouse A, Tennant A, Chamberlain M: **The impact of rheumatoid arthritis in the workplace**. *British Journal of Therapy and Rehabilitation* 2001, **8**(9):342-347.

34. Goodacre LJ, Goodacre JA: **Factors influencing the beliefs of patients with rheumatoid arthritis regarding disease-modifying medication**. *Rheumatology* 2004, **43**(5):583-586.

35. Grant M: **Mothers with Arthritis, Child Care and Occupational Therapy: Insight through Case Studies**. *British Journal of Occupational Therapy* 2001, **64**(7):322-329.

36. Griffith J, Carr A: **What is the impact of early rheumatoid arthritis on the individual?** *Best practice & research Clinical rheumatology* 2001, **15**(1):77-90.

37. Gronning K, Lomundal B, Koksvik HS, Steinsbekk A: **Coping with arthritis is experienced as a dynamic balancing process. A qualitative study**. *Clinical rheumatology* 2011, **30**(11):1425-1432.

38. Hay MC, Cadigan RJ, Khanna D, Strathmann C, Lieber E, Altman R, McMahon M, Kokhab M, Furst DE: **Prepared patients: internet information seeking by new rheumatology patients**. *Arthritis and rheumatism* 2008, **59**(4):575-582.

39. Headland M: **Using a website containing patient narratives to understand people's experiences of living with arthritis**. *Journal of orthopaedic Nursing* 2006, **10**(2):106-112.

40. Sarah H, Zoë C, Margaret B, Karen K, Sue T, Denise P, Maggie H: **Patients' perceptions of fatigue in rheumatoid arthritis: Overwhelming, uncontrollable, ignored**. *Arthritis Care & Research* 2005, **53**(5):697-702.

41. Hirsh D, Clerehan R, Staples M, Osborne RH, Buchbinder R: **Patient assessment of medication information leaflets and validation of the Evaluative Linguistic Framework (ELF)**. *Patient Educ Couns* 2009, **77**(2):248-254.

42. Hofmann D, Ibrahim F, Rose D, Scott DL, Cope A, Wykes T, Lempp H: **Expectations of new treatment in rheumatoid arthritis: developing a patient‐generated questionnaire**. *Health Expectations : An International Journal of Public Participation in Health Care and Health Policy* 2015, **18**(5):995-1008.

43. Hooper H, Ryan S, Hassell A: **The role of social comparison in coping with rheumatoid arthritis: an interview study**. *Musculoskeletal Care* 2004, **2**(4):195-206.

44. Howden S, Jones D, Martin D, Nicol M: **Employment and chronic non-cancer pain: insights into work retention and loss**. *Work (Reading, Mass)* 2003, **20**(3):199-204.

45. Hwang EJ, Kim YH, Jun SS: **Lived experience of Korean women suffering from rheumatoid arthritis: a phenomenological approach**. *Int J Nurs Stud* 2004, **41**(3):239-246.

46. Iaquinta M, H Larrabee J: **Phenomenological Lived Experience of Patients With Rheumatoid Arthritis**, vol. 19; 2004.

47. Kett C, Flint J, Openshaw M, Raza K, Kumar K: **Self-management strategies used during flares of rheumatoid arthritis in an ethnically diverse population**. *Musculoskeletal Care* 2010, **8**(4):204-214.

48. Kristiansen TM, Primdahl J, Antoft R, Horslev-Petersen K: **Everyday life with rheumatoid arthritis and implications for patient education and clinical practice: a focus group study**. *Musculoskeletal Care* 2012, **10**(1):29-38.

49. Kristiansen TM, Primdahl J, Antoft R, Horslev-Petersen K: **It means everything: continuing normality of everyday life for people with rheumatoid arthritis in early remission**. *Musculoskeletal Care* 2012, **10**(3):162-170.

50. Kumar K, Gordon C, Barry R, Shaw K, Horne R, Raza K: **‘It’s like taking poison to kill poison but I have to get better’: A qualitative study of beliefs about medicines in Rheumatoid arthritis and Systemic lupus erythematosus patients of South Asian origin**. *Lupus* 2011, **20**(8):837-844.

51. Kumar K, Daley E, Khattak F, Buckley CD, Raza K: **The influence of ethnicity on the extent of, and reasons underlying, delay in general practitioner consultation in patients with RA**. *Rheumatology* 2010, **49**(5):1005-1012.

52. Lacaille D, White MA, Backman CL, Gignac MA: **Problems faced at work due to inflammatory arthritis: new insights gained from understanding patients' perspective**. *Arthritis and rheumatism* 2007, **57**(7):1269-1279.

53. Lambert BL, Butin DN, Moran D, Zhao SZ, Carr BC, Chen C, Kizis FJ: **Arthritis care: comparison of physicians' and patients' views**. *Seminars in arthritis and rheumatism* 2000, **30**(2):100-110.

54. Larsson I, Bergman S, Fridlund B, Arvidsson B: **Patients' dependence on a nurse for the administration of their intravenous anti-TNF therapy: A phenomenographic study**. *Musculoskeletal Care* 2009, **7**(2):93-105.

55. Lempp H, Hofmann D, Hatch SL, Scott DL: **Patients' views about treatment with combination therapy for rheumatoid arthritis: a comparative qualitative study**. *BMC Musculoskelet Disord* 2012, **13**:200.

56. Lempp H, Scott D, Kingsley G: **The personal impact of rheumatoid arthritis on patients' identity: a qualitative study**. *Chronic Illness* 2006, **2**(2):109-120.

57. Li LC, Adam PM, Backman CL, Lineker S, Jones CA, Lacaille D, Townsend AF, Yacyshyn E, Yousefi C, Tugwell P *et al*: **Proof-of-concept study of a Web-based methotrexate decision aid for patients with rheumatoid arthritis**. *Arthritis Care Res (Hoboken)* 2014, **66**(10):1472-1481.

58. Li L, Townsend A, Adam P, Cox S, Amarsi Z, Backman C: **Crossing the threshold: Help-seeking for early symptoms in people with rheumatoid arthritis**. *Arthritis and Rheumatology* 2009, **60**.

59. Lindblad AK, Hartzema AG, Jansson L, Feltelius N: **Patients' views of priority setting for new medicines. A qualitative study of patients with rheumatoid arthritis**. *Scand J Rheumatol* 2002, **31**(6):324-329.

60. Linden C, Bjorklund A: **Living with rheumatoid arthritis and experiencing everyday life with TNF-alpha blockers**. *Scandinavian journal of occupational therapy* 2010, **17**(4):326-334.

61. Locker D: **Disability and disadvantage: the consequences of chronic illness**. London: Tavistock Publications; 1983.

62. Lorish CD, Richards B, Brown S, Jr.: **Perspective of the patient with rheumatoid arthritis on issues related to missed medication**. *Arthritis care and research : the official journal of the Arthritis Health Professions Association* 1990, **3**(2):78-84.

63. Lutze U, Archenholtz B: **The impact of arthritis on daily life with the patient perspective in focus**. *Scand J Caring Sci* 2007, **21**(1):64-70.

64. Mancuso CA, Paget SA, Charlson ME: **Adaptations made by rheumatoid arthritis patients to continue working: a pilot study of workplace challenges and successful adaptations**. *Arthritis care and research : the official journal of the Arthritis Health Professions Association* 2000, **13**(2):89-99.

65. Markusse IM, Akdemir G, Huizinga TW, Allaart CF: **Drug-free holiday in patients with rheumatoid arthritis: a qualitative study to explore patients' opinion**. *Clinical rheumatology* 2014, **33**(8):1155-1159.

66. Marshall NJ, Wilson G, Lapworth K, Kay LJ: **Patients' perceptions of treatment with anti-TNF therapy for rheumatoid arthritis: a qualitative study**. *Rheumatology* 2004, **43**(8):1034-1038.

67. McArthur MA, Birt L, Goodacre L: **"Better but not best": a qualitative exploration of the experiences of occupational gain for people with inflammatory arthritis receiving anti-TNFalpha treatment**. *Disabil Rehabil* 2015, **37**(10):854-863.

68. McPherson PBWJTHKMKM: **Living with arthritis—what is important?** *Disability and Rehabilitation* 2001, **23**(16):706-721.

69. McPherson KM, Brander P, Taylor WJ, McNaughton HK: **Consequences of stroke, arthritis and chronic pain—are there important similarities?** *Disability and Rehabilitation* 2004, **26**(16):988-999.

70. Meade T, Sharpe L, Hallab L, Aspanell D, Manolios N: **Navigating motherhood choices in the context of rheumatoid arthritis: women's stories**. *Musculoskeletal Care* 2013, **11**(2):73-82.

71. Meyfroidt S, Van der Elst K, De Cock D, Joly J, Westhovens R, Hulscher M, Verschueren P: **Patient experiences with intensive combination-treatment strategies with glucocorticoids for early rheumatoid arthritis**. *Patient Educ Couns* 2015, **98**(3):384-390.

72. Minnock P, Ringner A, Bresnihan B, Veale D, FitzGerald O, McKee G: **Perceptions of the Cause, Impact and Management of Persistent Fatigue in Patients with Rheumatoid Arthritis Following Tumour Necrosing Factor Inhibition Therapy**. *Musculoskeletal Care* 2017, **15**(1):23-35.

73. Mitton DL, Treharne GJ, Hale ED, Williams RA, Kitas GD: **The health and life experiences of mothers with rheumatoid arthritis: a phenomenological study**. *Musculoskeletal Care* 2007, **5**(4):191-205.

74. Moss P: **Negotiating spaces in home environments: Older women living with arthritis**. *Social Science & Medicine* 1997, **45**(1):23-33.

75. Neill J: **Transcendence and transformation in the life patterns of women living with rheumatoid arthritis**. *Advances in Nursing Science* 2002, **24**(4):27.

76. Nilsson I, Fitinghoff H, Lilja M: **Continuing to work after the onset of rheumatoid arthritis**. *Work (Reading, Mass)* 2007, **28**(4):335-342.

77. Nota I, Drossaert CH, Taal E, van de Laar MA: **Patients' considerations in the decision-making process of initiating disease-modifying antirheumatic drugs**. *Arthritis Care Res (Hoboken)* 2015, **67**(7):956-964.

78. Nyman CS, Lutzen K: **Caring needs of patients with rheumatoid arthritis**. *Nursing science quarterly* 1999, **12**(2):164-169.

79. O'Hare R, Muir A, Chapman S, Watson A, Hudson SA: **Identification of the pharmaceutical care issues of rheumatoid arthritis patients in secondary care**. *Pharmacy World and Science* 2001, **23**(5):183-184.

80. Oliver S, Bosworth A, Airoldi M, Bunyan H, Callum A, Dixon J, Home D, Lax I, O'Brien A, Redmond A *et al*: **Exploring the healthcare journey of patients with rheumatoid arthritis: a mapping project - implications for practice**. *Musculoskeletal Care* 2008, **6**(4):247-266.

81. Pasma A, van 't Spijker A, Luime JJ, Walter MJ, Busschbach JJ, Hazes JM: **Facilitators and barriers to adherence in the initiation phase of Disease-modifying Antirheumatic Drug (DMARD) use in patients with arthritis who recently started their first DMARD treatment**. *J Rheumatol* 2015, **42**(3):379-385.

82. Ruth P: **Bringing back the body without the blame?: the experience of ill and disabled people at work**. *Sociology of Health & Illness* 1995, **17**(5):605-631.

83. Plach SK, Stevens PE, Moss VA: **Social Role Experiences of Women Living with Rheumatoid Arthritis**. *Journal of Family Nursing* 2004, **10**(1):33-49.

84. Plach S, E Stevens P, A Moss V: **Corporeality: Women’s Experiences of a Body With Rheumatoid Arthritis**, vol. 13; 2004.

85. Radford S, Carr M, Hehir M, Davis B, Robertson L, Cockshott Z, Tipler S, Hewlett S: **‘It's quite hard to grasp the enormity of it’: Perceived needs of people upon diagnosis of rheumatoid arthritis**. *Musculoskeletal Care* 2008, **6**(3):155-167.

86. Rao J, Rhonda R, Kim K, weinberger M: **Using focus groups to understand arthritis patients' perceptions about unconventional therapy**. *Arthritis & Rheumatism* 1998, **11**(4):253-260.

87. Rice GE, Young LH: **A folk model of arthritis**. *Health Values: The Journal of Health Behavior, Education & Promotion* 1994.

88. Rose G: **Why do patients with rheumatoid arthritis use complementary therapies?** *Musculoskeletal Care* 2006, **4**(2):101-115.

89. Ryan S, Hassell A, Dawes P, Kendall S: **Control perceptions in patients with rheumatoid arthritis: the impact of the medical consultation**. *Rheumatology* 2003, **42**(1):135-140.

90. Ryan S, Hassell A: **Perceptions of control in patients with rheumatoid arthritis**. *Nursing Times* 2003, **99**:36-38.

91. Sakalys JA: **Illness behavior in rheumatoid arthritis**. *Arthritis care and research : the official journal of the Arthritis Health Professions Association* 1997, **10**(4):229-237.

92. Salt E, Peden A: **The complexity of the treatment: the decision-making process among women with rheumatoid arthritis**. *Qual Health Res* 2011, **21**(2):214-222.

93. Salt E, Rowles GD, Reed DB: **Patient's perception of quality patient--provider communication**. *Orthopedic nursing* 2012, **31**(3):169-176.

94. Sanderson T, Calnan M, Morris M, Richards P, Hewlett S: **The impact of patient-perceived restricted access to anti-TNF therapy for rheumatoid arthritis: a qualitative study**. *Musculoskeletal Care* 2009, **7**(3):194-209.

95. Sanderson T, Calnan M, Morris M, Richards P, Hewlett S: **Shifting normalities: interactions of changing conceptions of a normal life and the normalisation of symptoms in rheumatoid arthritis**. *Sociol Health Illn* 2011, **33**(4):618-633.

96. Sanderson T, Hewlett S, Richards P, Morris M, Calnan M: **Utilizing qualitative data from nominal groups: exploring the influences on treatment outcome prioritization with rheumatoid arthritis patients**. *J Health Psychol* 2012, **17**(1):132-142.

97. Sanderson T, Morris M, Calnan M, Richards P, Hewlett S: **What outcomes from pharmacologic treatments are important to people with rheumatoid arthritis? Creating the basis of a patient core set**. *Arthritis Care Res (Hoboken)* 2010, **62**(5):640-646.

98. Sanderson T, Morris M, Calnan M, Richards P, Hewlett S: **'It's this whole picture, this well-being': patients' understanding of 'feeling well' with rheumatoid arthritis**. *Chronic Illn* 2010, **6**(3):228-240.

99. Sandhu S, Veinot P, Embuldeniya G, Brooks S, Sale J, Huang S, Zhao A, Richards D, Bell MJ: **Peer-to-peer mentoring for individuals with early inflammatory arthritis: feasibility pilot**. *BMJ Open* 2013, **3**(3).

100. Schildmann J, Grunke M, Kalden JR, Vollmann J: **Information and participation in decision-making about treatment: a qualitative study of the perceptions and preferences of patients with rheumatoid arthritis**. *Journal of medical ethics* 2008, **34**(11):775-779.

101. Schneider M, Manabile E, Tikly M: **Social aspects of living with rheumatoid arthritis: a qualitative descriptive study in Soweto, South Africa – a low resource context**. *Health and Quality of Life Outcomes* 2008, **6**(1):54.

102. Shariff F, Carter J, Dow C, Polley M, Salinas M, Ridge D: **Mind and Body Management Strategies for Chronic Pain and Rheumatoid Arthritis**. *Qualitative Health Research* 2009, **19**(8):1037-1049.

103. Shaul MP: **Transitions in chronic illness: rheumatoid arthritis in women**. *Rehabil Nurs* 1997, **22**(4):199-205.

104. P. Shaul M: **From early twinges to mastery: The process of adjustment in living with rheumatoid arthritis**, vol. 8; 1995.

105. Sheppard J, Kumar K, Buckley C, Shaw K, Raza K: **‘I just thought it was normal aches and pains’: a qualitative study of decision-making processes in patients with early rheumatoid arthritis**. *Rheumatology* 2008, **47**(10):1577–1582.

106. Sinclair VG, Blackburn DS: **Adaptive coping with rheumatoid arthritis: the transforming nature of response shift**. *Chronic Illn* 2008, **4**(3):219-230.

107. Stamm TA, Machold KP, Smolen J, Prodinger B: **Life stories of people with rheumatoid arthritis who retired early: how gender and other contextual factors shaped their everyday activities, including paid work**. *Musculoskeletal Care* 2010, **8**(2):78-86.

108. Tanja S, Jon W, Klaus M, Gaynor S, Josef S: **Occupational balance of women with rheumatoid arthritis: a qualitative study**. *Musculoskeletal Care* 2004, **2**(2):101-112.

109. Stamm T, Lovelock L, Stew G, Nell V, Smolen J, Jonsson H, Sadlo G, Machold K: **I Have Mastered the Challenge of Living With a Chronic Disease: Life Stories of People With Rheumatoid Arthritis**. *Qualitative Health Research* 2008, **18**(5):658-669.

110. Stenström CH, Bergman B, Dahlgren LO: **Everyday life with rheumatoid arthritis: A phenomenographic study**. *Physiotherapy Theory and Practice* 1993, **9**(4):235-243.

111. Stephens M, Yoshida K: **Independence and autonomy among people with rheumatoid arthritis**. *Can J Rehabil* 1999, **12**:229-235.

112. Stockdale J, Goodacre L: **'It's magic stuff': the experiences of patients with ankylosing spondylitis taking anti-TNF-alpha medication**. *Musculoskeletal Care* 2009, **7**(3):162-177.

113. Jennifer S, James S, Hazel R: **An Exploration of the Impact of Anti‐TNFα Medication on Exercise Behaviour in Patients with Ankylosing Spondylitis**. *Musculoskeletal Care* 2014, **12**(3):150-159.

114. Townsend A, Backman CL, Adam P, Li LC: **A qualitative interview study: patient accounts of medication use in early rheumatoid arthritis from symptom onset to early postdiagnosis**. *BMJ Open* 2013, **3**(2).

115. Townsend A, Adam P, Cox SM, Li LC: **Everyday ethics and help-seeking in early rheumatoid arthritis**. *Chronic Illn* 2010, **6**(3):171-182.

116. van der Elst K, Meyfroidt S, De Cock D, De Groef A, Binnard E, Moons P, Verschueren P, Westhovens R: **Unraveling Patient-Preferred Health and Treatment Outcomes in Early Rheumatoid Arthritis: A Longitudinal Qualitative Study**. *Arthritis Care Res (Hoboken)* 2016, **68**(9):1278-1287.

117. Van der Meer M, Hoving JL, Vermeulen MI, Herenius MM, Tak PP, Sluiter JK, Frings-Dresen MH: **Experiences and needs for work participation in employees with rheumatoid arthritis treated with anti-tumour necrosis factor therapy**. *Disabil Rehabil* 2011, **33**(25-26):2587-2595.

118. van Tuyl LH, Plass AM, Lems WF, Voskuyl AE, Kerstens PJ, Dijkmans BA, Boers M: **Discordant perspectives of rheumatologists and patients on COBRA combination therapy in rheumatoid arthritis**. *Rheumatology (Oxford)* 2008, **47**(10):1571-1576.

119. van Tuyl LH, Hewlett S, Sadlonova M, Davis B, Flurey C, Hoogland W, Kirwan J, Sanderson T, van Schaardenburg D, Scholte-Voshaar M *et al*: **The patient perspective on remission in rheumatoid arthritis: 'You've got limits, but you're back to being you again'**. *Ann Rheum Dis* 2015, **74**(6):1004-1010.

120. Varekamp I, Haafkens JA, Detaille SI, Tak PP, van Dijk FJ: **Preventing work disability among employees with rheumatoid arthritis: what medical professionals can learn from the patients' perspective**. *Arthritis and rheumatism* 2005, **53**(6):965-972.

121. Ward V, Hill J, Hale C, Bird H, Quinn H, Thorpe R: **Patient priorities of care in rheumatology outpatient clinics: A qualitative study**, vol. 5; 2007.

122. Wiener CL: **The burden of rheumatoid arthritis: Tolerating the uncertainty**. *Social Science & Medicine (1967)* 1975, **9**(2):97-104.

123. Williams B BJ: **Falling out with my shadow: lay perceptions of the body in the context of arthritis**. In: *The body in everyday life.* edn. Edited by Nettleton S WJ. London: Routledge; 1998: 124-141.

124. Williams AE, Graham AS: **'My feet: visible, but ignored . . .' A qualitative study of foot care for people with rheumatoid arthritis**. *Clinical rehabilitation* 2012, **26**(10):952-959.

125. Williams G, H.N. Wood P: **Coming to terms with chronic illness: The negotiation of autonomy in rheumatoid arthritis**, vol. 10; 1988.

126. Williams G: **The genesis of chronic illness: narrative re-construction**. *Sociol Health Illn* 1984, **6**(2):175-200.

127. Yoshida K: **Uncertainty in the lives of people with spinal cord injury and rheumatoid arthritis**. *Canadian Journal of rehabilitation* 1996.

128. Zhang J, Verhoef MJ: **Illness management strategies among Chinese immigrants living with arthritis**. *Soc Sci Med* 2002, **55**(10):1795-1802.
